# Protective Effect of Thyme Honey against Valproic Acid Hepatotoxicity in Wistar Rats

**DOI:** 10.1155/2021/8839898

**Published:** 2021-02-20

**Authors:** Rezvan Omidipour, Leila Zarei, Mandana Beigi Boroujeni, Asghar Rajabzadeh

**Affiliations:** ^1^Department of Anatomy Sciences, Faculty of Medicine, Lorestan University of Medical Sciences, Khorramabad, Iran; ^2^Razi Herbal Medicines Research Center, Lorestan University of Medical Sciences, Khorramabad, Iran

## Abstract

**Introduction:**

Valproic acid is a medication most commonly used in the treatment of emotional and neurological depression, psychological imbalances, epilepsy, and bipolar disorder. Dark honey, like thyme honey, contains more antioxidant compounds than other samples. The purpose of this study was to evaluate the effect of thyme honey on the potential hepatic effects of valproic acid.

**Methods:**

In this study, 48 male rats were randomly divided into 8 groups (*n* = 6): G1 (control): healthy rats (normal saline 0.9%), G2: thyme honey (1 g/kg), G3: thyme honey (2 g/kg dose), G4: thyme honey (3 g/kg dose), G5: VPA (500 mg/kg), G6: VPA (500 mg/kg) and thyme honey (1 g/kg), G7: VPA (500 mg/kg) and thyme honey (2 g/kg dose), and G8: VPA (500 mg/kg) and thyme honey (3 g/kg dose). Groups G1 to G5 received the drug for 28 days. On day 14, administration of thyme honey for G6 to G8 groups was carried out using gavage until day 28. VPA was administered one hour after honey. To carry out the biochemical evaluation, blood samples were collected from all the groups and their serums were used for MDA, TAC, and liver enzymes (AST, ALT, and GGT). Tissue samples of each rat were also removed for histological studies with hematoxylin-eosin and Masson's trichrome staining.

**Results:**

The use of thyme honey significantly improved the histopathological parameters of the liver tissue, including hypertrophic degeneration and nucleus alteration, expansion of sinusoids, fibrosis and hepatic necrosis, and inflammation as well as hypertrophy of Kupffer cells. In the groups receiving VPA, the rate of lipid peroxidation increased, which indicates the destruction of the liver cell membrane due to drug consumption. TAC levels also increased following increase in thyme honey dosage (*p* ≤ 0.05). The results of liver enzyme analysis showed a decrease in AST and ALT levels in the G6 group and a decrease in GGT level in the G8 group (*p* ≤ 0.05).

**Conclusion:**

Based on the results of this study, it seems that high percentage of antioxidants in thyme honey enabled it to improve hepatic complications and reduce the rate of hepatocellular destruction.

## 1. Introduction

Valproic acid and sodium valproate, with the brand names Depakin and Valpakin, are antiepileptic agents that can control bipolar disorder [1]. Based on the mechanism of action of VPA, by increasing GABA blood circulation and increasing nerve synapses, it suppresses the additional stimulation of neurons in the brain [2]. Side effects of VPA include tremors and nausea, stomach pain, obesity, swelling in the legs, hair loss [2], lethal hepatotoxicity (production of toxic metabolites and induction of allergic immune reactions) [3] and teratogenicity (increasing neural tube defects), lip and cleft palate, cardiovascular abnormalities, genital defects, growth retardation, endocrine disorders, limb defects, and autism [4]. One of the most important complications of VPA is fetal liver toxicity, which leads to liver failure syndrome and mitochondrial dysfunction [5]. After induction damage, in vivo or in vitro, levels of liver enzymes (ALP, AST, ALT, LDH, and GGT) increase [2]. Antioxidants protect the cells against free radical damage. Plant antioxidants, including phenols, are involved in regulating the activity of liver enzymes. These antioxidants reduce cellular damage caused by oxidants or inflammatory cytokines [6]. In fact, these physiological compounds are used to limit oxidative stress [5, 7]. Oxidative stress in biological systems is defined as an imbalance in the prooxidant/antioxidant system. Oxidants are mainly composed of reactive oxygen species (ROS) and reactive nitrogen species (RNS) which are active species with detrimental effects on organs. Studies on animal models have suggested that VPA treatment is associated with oxidative stress, an elevation in the liver level of an endogenous lipid peroxidation marker, and mitochondrial dysfunction. Therefore, there is no generic opinion about the side effects of VPA on liver biomarkers and histological structure. Toxic effects of VPA on cell lines as well as its hepatotoxicity have called attention to the study of the inhibitory role of natural compounds such as domestic antioxidants against VPA and other chemical drugs.

Dark honey, like thyme honey, contains more antioxidant compounds than other species, including phenolic compounds and flavonoids [7]. Thyme is a member of the nectar-producing mint family, Lamiaceae. Thyme honey, which is produced from different species (*Thymus* spp.), is very effective in treating liver damage [7, 8].

### 1.1. Objectives

Since the clinical dose of valproate leads to liver failure syndrome and mitochondrial dysfunction, studies between natural antioxidants and chemical drugs can contribute to the reduction of side effects in patients. Hence, the aim of this study was to investigate the effect of thyme honey on liver complications after valproic acid consumption in male Wistar rats.

## 2. Material and Methods

### 2.1. Honey Samples

The honey sample was purchased from the reputable company Meda (Beekeeping Cooperative of “Nahl Mihan,” Alborz Province, Iran) and kept at 5°C until testing (at high temperatures, it loses its properties, and at low temperatures, it freezes (.To measure the antioxidant capacity of honey, spectrophotometry was performed.

### 2.2. Total Antioxidant Content (TAC) Test

The TAC method is a method in which the total antioxidant capacity of a honey sample is calculated. For this purpose, 999 micrograms of the honey extract were combined with 999 micrograms of reagent (including 6.9 micron sulfuric acid, 18 ml micron sodium phosphate, and 8 ml micron ammonium molybdenum). They were placed in a bain-marie for 19 minutes. After cooling, the absorbance of the samples was read at a wavelength of 611 nm at specified times. The read values were calculated after being placed in the TAC standard formula. The solution of ascorbic acid and quercetin was used as a standard, and the antioxidant capacity of honey was expressed as equivalent to ascorbic acid and quercetin. Therefore, the total antioxidant capacity of the thyme honey sample was obtained [9]. The equivalent amount of 9998 nmol of ascorbic acid per gram of honey was calculated. This value indicates the amount of total antioxidants in the honey sample, which is calculated based on the standard diagram of ascorbic acid.

### 2.3. Test of Total Flavonoid Content

The amount of flavonoids was measured by aluminum chloride colorimetry. In this method, which was carried out manually, 511 microliters of the honey extract was combined with 511 microliters of AlCl_3_ 2%, after 1 hour at room temperature. Subsequently, the absorption was read during the 421 nm wave. The total amount of flavonoids in honey is expressed as milligrams equivalent to quercetin. Standard quantities are plotted based on quercetin concentrations. The amounts read after being included in the standard formula were calculated [10]. This amount is equivalent to 7.38 milligrams of quercetin per gram of honey. This value represents the total flavonoid content in the honey sample, which is calculated based on the standard quercetin diagram.

### 2.4. DPPH Test

The method of evaluating the antioxidant capacity with DPPH (2,2-diphenyl-1-picrylhydrazyl) radical is a method based on the reduction of free radicals. As free radicals are reduced by the antioxidants found in thyme honey, the recorded wavelength in the device is determined. Only one type of antioxidant is used in this assay, which is considered the basis for measurement. Radical inhibition of DPPH is the basis for evaluating antioxidant capacity. Combine 111 microliters of the honey extract with 51 microliters of Fulcium cocaltive reagent, place it at room temperature for 5 minutes, and recombine it with 751 microliters of 6% sodium carbonate and then vortex it. After being at room temperature for 41 minutes, absorption at wavelength 725 nm was read. The readings were calculated after being placed in the standard formula of gallic acid [11]. This value was calculated as 11.23 mg gallic acid per gram of honey.

### 2.5. Animals and Study Groups

In this study, 48 male Wistar rats (weighed 250 ± 10 g, ages 50 ± 5 days) were randomly classified into eight groups (*n* = 6). The animals were purchased from the Razi Herbal Medicines Research Center, Lorestan University of Medical Sciences. In order to adapt the animals to the environment, all the groups were kept in standard conditions: temperature of 25 ± 2°C, relative humidity of 50 ± 10%, light-dark cycle of 10/14 hours, clean beds, adequate water, and special mice's dry food.

(1) $$\begin{array}{c} \text{Group G}1\;\left(\text{sham}\right)\text{: control rats }\left(\text{normal saline }0.9\%,\text{intraperitoneally}\right)\\ \text{Group G}2:\text{receiving TH }\left(1\text{ g}/\text{kg}\right)\\ \text{Group G}3:\text{receiving TH }\left(2\text{ g}/\text{kg}\right)\\ \text{Group G}4:\text{receiving TH }\left(3\text{ g}/\text{kg}\right)\\ \text{Group G}5:\text{receiving VPA }\left(500\text{ mg}/\text{kg}\right)\\ \text{Group G}6:\text{receiving TH }\left(1\text{ g}/\text{kg}\right)\text{ and VPA }\left(500\text{ mg}/\text{kg}\right)\\ \text{Group G}7:\text{receiving TH }\left(2\text{ g}/\text{kg}\right)\text{ and VPA }\left(500\text{ mg}/\text{kg}\right)\\ \text{Group G}8:\text{receiving TH }\left(3\text{ g}/\text{kg}\right)\text{ and VPA }\left(500\text{ mg}/\text{kg}\right) \end{array}$$

Multiple asterisks (∗∗∗) indicate that doses of VPA [12, 13] and TH [14] were selected based on references from previous studies.

An asterisk indicates (∗) indicates that samples of TH and VPA were dissolved in normal saline 0.9% received by the rats via gavage.

For 28 days, G1 received 1 cc of normal saline daily as an intraperitoneal injection [12]. Groups G2, G3, and G4 received TH in solution daily for 28 days. The VPA group received the drug in solution at a dose of 500 mg/kg for the first 14 days. The G6, G7, and G8 groups also received TH and VPA, based on the prescribed doses, in the second 14 days (VPA was given 1 hour after TH).

### 2.6. Ethical Consideration

This study was carried out in accordance with the recommendations for the care and the use of laboratory animals. The research project has received the confirmation of the institution's Ethics Committee (Lorestan University of Medical Sciences) with the number of IR LUMS.REC.1398.084.

### 2.7. Animal Test Method

The study was completed in 28 days, and after the completion of drug therapy, the samples were taken from the animals. The blood samples were taken from the heart of the animals anesthetized with inhaled chloroform. The blood samples were centrifuged at 10000 rpm at room temperature, and serum was collected in the top of the tubes and transferred to microtubes in order to be frozen. The samples of hepatic tissues were taken by using the sterile devices and were put in microtubes. Serums and tissues were labeled appropriately and then were kept at -70°C. A part of the tissues was placed in formalin 10% solution for histologic observations.

### 2.8. Hepatic Enzyme Test (ALT, AST, and GGT)

The level of hepatic enzymes was measured for evaluating the hepatic function. The kits that were used are from Pars Azmoon Co. (I. R. Iran). After defrosting, the serum samples were transferred to special cups. These cups were placed in their racks in a BT1000 autoanalyzer device. The levels of ALT, AST, and GGT enzymes were measured and recorded. These records were assessed according to standards. This process was repeated twice from the collected serums.

### 2.9. Measuring MDA and TAC

About 50-100 mg of the tissue sample was weighed, and 500 to 1000 ml lysing buffer was added to it according to the test protocol proposed by the kit manufacturer. With a glass homogenizer, we homogenized the sample and centrifuged it at 10000 rpm for 10 minutes. The supernatant fluid was separated and applied as the test sample. The procedure was completed according to the kits' protocols, and test results were recorded. These measurements were reported based on the standard formula of MDA and TAC with an ELISA reader. By placing the values obtained based on the following formula, the amount of MDA in the groups was obtained (Table 1).

Moreover, by placing the values obtained based on the following formula, the amount of TAC in the groups was obtained (Table 2).

### 2.10. Histological Studies

A piece of liver was removed from each rat in each group (6 pieces from each group), and then, it was cleaned and placed in a physiological NaCl solution for 30 minutes. Subsequently, the sample was placed in a fixative solution (formalin 10%) for 24 hours. For dehydrating, the specimen was passed through alcohol solutions of 70%, 80%, and 95% and finally absolute alcohol. The sample was placed in xylol for 1 h for the replacement of alcohol by xylol. Then, the samples were immersed in melted paraffin (62°C) to make the xylol exit from the tissue and be replaced with paraffin. The cubic molds containing the specimens were filled with paraffin and after cooling were cut as serial section layers at the thickness of 5 microns. This activity was performed using a microtome. The layers were dewrinkled by floating them on the surface of warm water. The cuttings were passed through descending concentrations of xylol and alcohol. We used two methods of staining, including the H&E method and Masson's trichrome technique. This process was repeated three times, and 10 sections with suitable quality were obtained from every group of each rat.

### 2.11. Statistical Analysis

The results are presented as the mean values ± standard deviations (SD). The analysis of liver enzyme data levels was completed by using SAS ver. 6.2, and histochemical data levels were completed by using Microsoft Excel software. The statistical analyses of the biochemical data were conducted using Tukey's test. The variance in level of *p* < 0.05 was designated as significant.

## 3. Results

### 3.1. Histopathology

**Figure 1:**
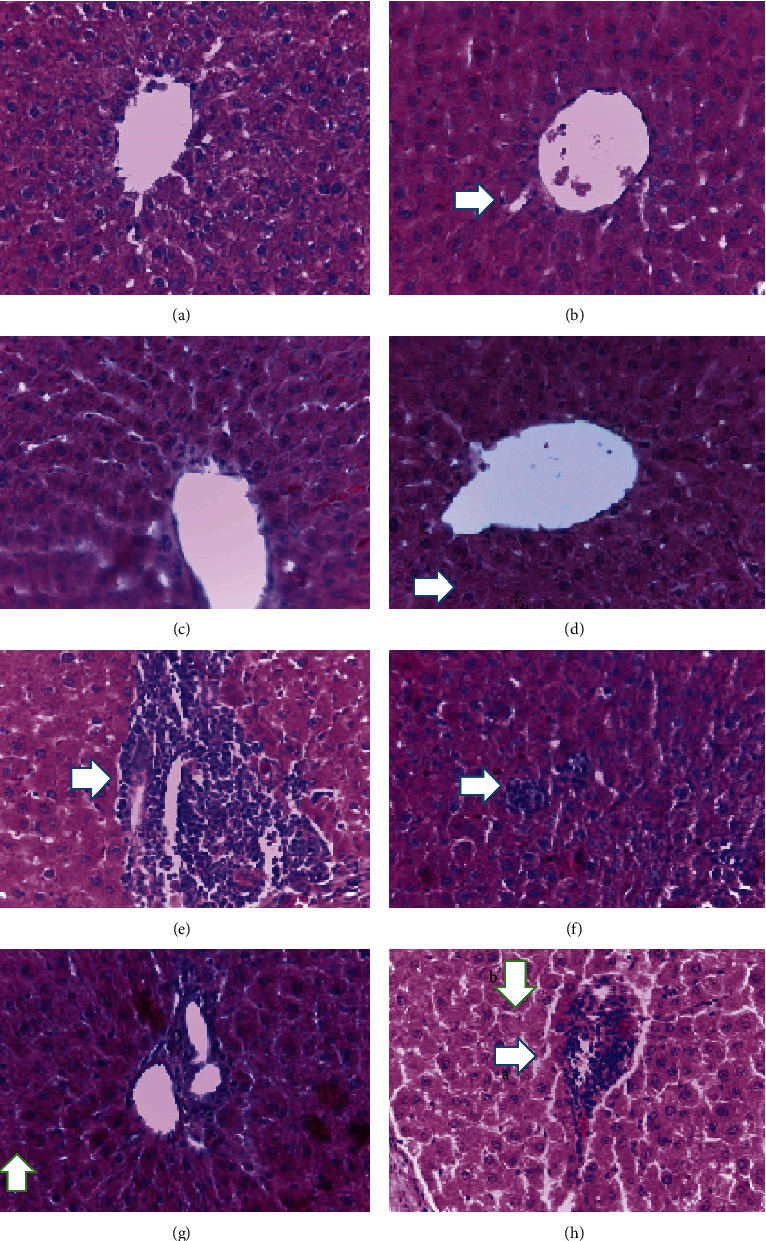
The control groups G1 (a), G2 (b), G3 (c), and G4 (d) (in (b), the arrow shows the sinusoid; in (d), the arrow points to the hepatocyte) (100x). (e) a: accumulation of inflammatory cells; b: changes in hepatocytes' nucleus. (f) Some remaining inflammation. (g) Settlement of inflammation and apoptosis. (h) Elevated inflammation ratios (arrows: a—accumulation of inflammatory cells; b—changes in nucleus size and hepatocytes' cytoplasm). H&E, 40x, scale bar: 50 *μ*m.

**Figure 2:**
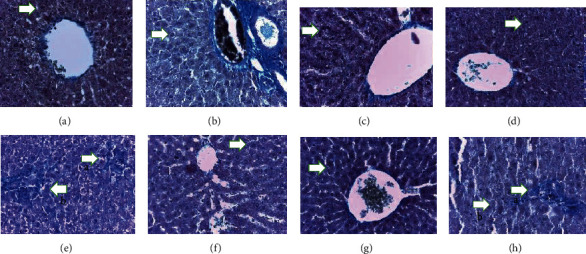
The control groups G1 (a), G2 (b), G3 (c), and G4 (d). In these groups, hepatocytes are normal in shape (the arrow points to normal hepatocytes) (100x). (e) Arrows: a—accumulation of inflammatory cells; b—expanding sinusoid space. (f) Arrows: a and b—inflammation. (g) Arrow: b—settlement of inflammation and apoptosis in the G7 group. (h) Arrows: a—inflammatory cell aggregate; b—changes in nucleus size and hepatocytes' cytoplasm. Masson's trichrome (40x) scale bar: 20 *μ*m.

After staining, the samples were evaluated by using a light microscope with different magnifications. According to histologic studies, the control group had central normal veins, sinusoids, and hepatocytes. Similar to the control group, groups only receiving TH (G2, G3, and G4) had normal hepatocytes and normal hepatic tissue spaces (Figures 1 and 2). Based on Masson's trichrome staining, the rats that received TH showed traces of collagen fibers around the port space compared to the control group.

In comparison to the control group, in G5, there were significant alterations, including accumulation of inflammatory cells, expansion of the sinusoid spaces, and changes in nucleus size as well as hepatocytes' cytoplasm. The microscopic observation in G6 (1 g TH+VPA) revealed some inflammations and morphologic alterations in hepatocytes compared to the control group. In G7 (2 g TH+VPA), greater reductions in inflammation were observed. However, in G8 (3 g TH+VPA), the inflammation was at high levels similar to that in G5 (Figure 1).

### 3.2. Histologic Scoring Outcomes

Histological scores have been evaluated in studies of tissue samples by using optical microscopes. This evaluation method helps us score the differences between the study groups based on the subjects. In histological scoring, we investigated the changes in hepatic cells (hypertrophic degeneration and nucleus alterations), expansion of hepatic sinusoids, hepatic fibrosis and necrosis, inflammation of hepatocytes, and hypertrophy of Kupffer cells [12] (Table 3).

According to the results of the present study, the highest rate of hepatocyte changes (hypertrophic degeneration and nuclear changes, hepatic sinusoidal dilation, hepatic fibrosis and necrosis, hepatocyte inflammation, and Kupffer cell hypertrophy) was observed in G5 and the lowest in G1 and G2. In G7, hepatic fibrosis and necrosis, inflammation, and other signs were observed lowly compared to those in G5, G6, and G8. It appears that hepatic changes are going to improve in G7 with TH 2 g/kg dosage. Nevertheless, TH (1 g and 3 g dosages) could not improve the changes. According to the outcomes, it is obvious that a special dosage of TH can reduce hepatic complications. Moreover, incorrect treatment with natural antioxidants could be poisonous.

### 3.3. Biochemistry

#### 3.3.1. MDA and TAC

**Figure 3:**
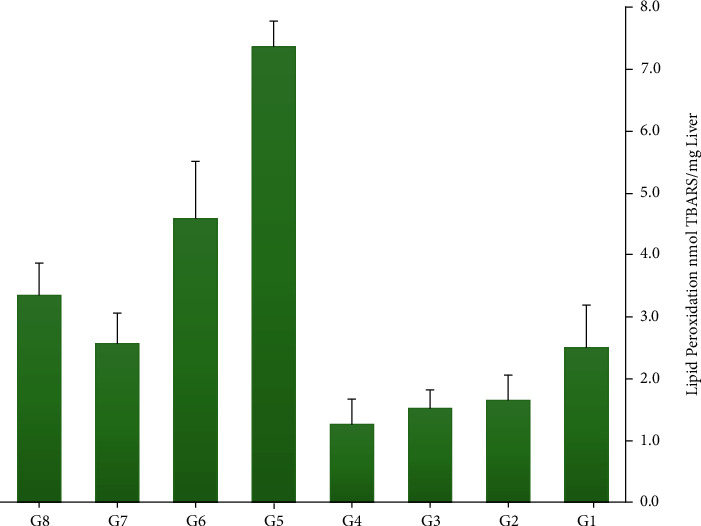
A comparison of MDA levels in the groups (the highest level was related to G5, and the lowest level was related to G4).

Biochemical tests for MDA and TAC on the homogenized tissues of the liver have reported the following results. The ELISA reader measurements show lipid peroxidation (MDA) in hepatic tissue after VPA administration and treatment with TH by doses of 1 to 3 grams. The rate of peroxidation is higher in G1 compared to G2, G3, and G4. This amount decreases in the three groups that received only honey with increasing doses, respectively. In G5, MDA level are more than that in other groups mostly in comparison to G4. MDA level changes in these groups show a significant reduction after TH utilization and a notable increase by VPA administration. In other groups, MDA level compared to that in G5 presented slow decreases. In group G7, the levels of MDA are less than those in groups G5, G6, and G8, which indicates the greatest effect of honey against VPA in this dose. The findings show that MDA in groups taking TH without VPA (except group G4) reveals no significant differences with the control group (*p* > 0.05) (Figure 3).

**Figure 4:**
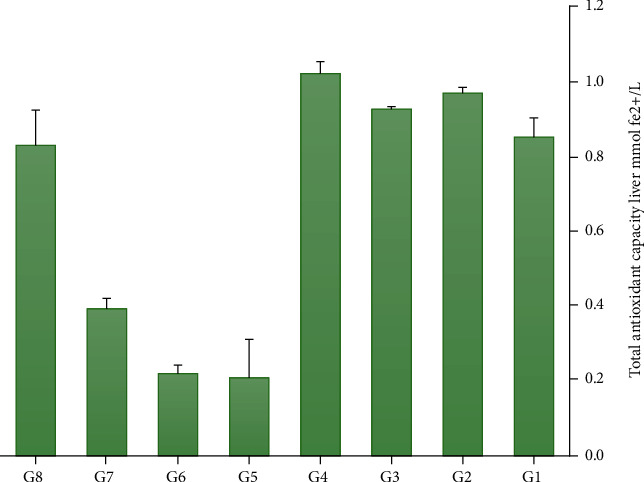
A comparison of TAC levels in the groups (the highest level was related to G4, and the lowest level was related to G5).

Figure 4 shows the TAC measurement in the liver tissue after VPA administration and treatment with 1, 2, and 3 g TH. The results suggest that the TAC level in the groups receiving TH without VPA has no significant difference with the control group (*p* > 0.05). The level of TAC in the G1 group is slightly lower than that in G2, G3, and G4 groups. In the last three groups, this level is high, particularly in G4, which received the highest dose of honey. In G6, G7, and G8 groups, consumption of TH (with increasing dose) was able to increase the TAC level. It was observed that TAC level significantly decreased in the G5 group (VPA only) compared to the control group. In the TH plus VPA groups (G6, G7, and G8), low levels of TAC were compensated based on the increasing dose of TH, particularly in G8. Therefore, the highest and lowest levels of TAC are related to G4 and G5, respectively (*p* < 0.05). It seems that raising TH dose can increase the TAC level (Figure 4).

### 3.4. Hepatic Enzymes (AST, ALT, and GGT)

**Figure 5:**
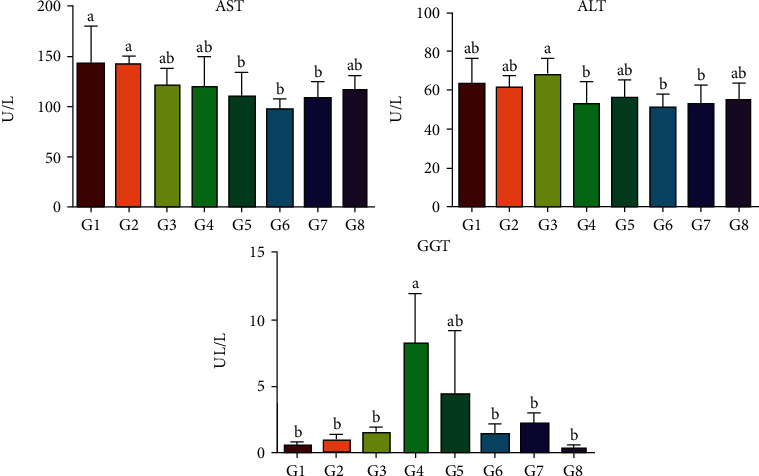
A comparison of hepatic enzyme (AST, ALT, and GGT) values in the groups (a and b: dissimilar letters in each row indicate a significant difference between the groups.)

The results suggest that AST levels are significantly different between the groups (*p* < 0.05). In G1 and G2 groups, AST is almost equal without a significant difference, while in G3 and G4 groups, this amount has decreased compared to G1 and G2 groups. In the G5 group, the amount of enzyme was less than that in the first four groups (*p* < 0.05). In TH plus VPA groups, increasing the TH dose shows a suitable outcome, mostly in G8 (3 g dose). TH (1 g dose) did not show supplementary effects against VPA (Figure 5).

The levels of ALT enzyme present significant differences and confusing outcomes among the groups (*p* < 0.05). The level of ALT in G1 is slightly higher than that in all the groups except G3. In G3, this amount is higher than that in the control and other groups. ALT level in G5 compared to G4, G6, G7, and G8 shows notable differences (*p* < 0.05). In groups G6, G7, and G8, the level of ALT enzyme increased, respectively. In the G6 group, 1 g of TH plus VPA probably had a greater effect on the ALT enzyme than that in other groups. Finally, after analyzing the ALT level between the groups, it appears that the current enzyme can show different aspects among healthy and treated animals (Figure 5).

The findings related to GGT showed significant differences among the groups (*p* < 0.05). The amount of GGT in G1 is less than that in all the groups except G8. In G4, this level has reached its highest level among the groups. In G5, the level of enzyme is higher than that in the control and other groups except G4. In the groups treated with TH and VPA (G6, G7, and G8), TH (2 g dose) could improve the side effects of VPA compared to that in G6 and G8. According to the data, it seems that GGT level was reduced based on the balance among VPA and TH consumption. Normally, TH (1 g) in G6 has been located into the second row after 3 g dose for neutralizing VPA effects. Showing various behaviors from TH with different doses might require further studies to find out the firm medical dose (*p* < 0.05) (Figure 5).

## 4. Discussion

Oxidative stress in biological systems is defined as an imbalance in the prooxidant/antioxidant system that leads to damage. Due to their toxic effects, ROS (reactive oxygen species) can lead to the peroxidation of membrane lipids and the production of MDA in the liver and other organs [4, 13]. In this study, particularly in the histopathology findings, it was proven that the use of VPA causes inflammation and increases oxidative stress in rat liver tissue. Thyme honey had a significant effect on the reduction of inflammation and oxidative stress caused by VPA in the liver.

Valproic acid (sodium valproate, divalproex) is one of the most common drugs that can prevent or control epilepsy and bipolar disorder. It is used alone or in combination with other medications to prevent migraine headache as well. One of the important cases of this drug is the possibility of developing liver failure, which is especially at risk in children under two years of age and people who are genetically predisposed to this complication [13]. The combination of VPA with its oxidant properties can upset the antioxidant balance and result in liver damage [4]. In the animal category, VPA can accelerate degradation of liver tissue by altering the levels of liver enzymes, increasing the level of lipid peroxidation, reducing total antioxidant capacity, and histologically altering the cell structure [14]. There are two types of VPA-mediated hepatotoxicity, which are associated with inflammation, enzyme changes, and necrosis [14]. There is remarkable evidence showing that the matrix and intermembrane space (mitochondrial dysfunction) are the main sites of ROS generation, which can cause pathological condition. It seems that oxidative stress is the main reason for the toxicity of VPA treatment [15].

According to previous studies, idiosyncratic hepatotoxicity may occur after treatment with chemical drugs. However, it has been indicated in some studies that VPA-mediated hepatotoxicity is mainly based on dose-dependent factors. Similarly, in the present study, we observed type I (inflammation and enzyme changes) VPA-mediated hepatotoxicity following the administration 500 mg/kg dose. Inflammatory infiltration and fibrosis, which indicate liver damage, may not increase enzyme levels. In the study conducted by Shaaban and El-Agamy, significant increases in the levels of all three enzymes (AST, ALT, and ALP) were observed in rats treated with only 300 mg/kg VPA [16]. Similarly, Nazmy et al. showed that AST could increase after 700 mg/kg VPA in mice [17]. Li et al. who administered 100-500 mg/kg VPA did not show significant changes in liver enzyme levels [18]. In our study, changes in liver enzymes were also different. As mentioned in Results, ALT and AST levels in the control group were higher than those in the VPA group, and a certain dose of TH could modify the alterations. Consumption of TH alone did not have a significant effect on AST and ALT enzymes. In this case, it is hypothesized that the variety in the behavior of enzymes is related to the laboratory environment or animal species. With respect to some animal and human studies, it is obvious that long-term use of VPA may suggest it as a toxic drug with both reversible and irreversible hepatic failures via inducing or increasing oxidative stress markers [19]. The MDA level in the VPA group has reached the highest level compared to that in the control and other groups. Furthermore, the MDA level in the G4 group, which received the highest dose of honey, reached the lowest level, indicating the lowest rate of peroxidation and cell destruction in this group. It is proposed that elevated oxidative stress and decreased antioxidant enzyme activity (TAC) play pivotal roles in cellular death. It has been indicated in some studies that TAC level after the consumption of VPA depends on demographic properties and treatment protocols [20]. According to our findings, the amount of TAC in the G4 group, which received a dose of 3 grams of honey alone, was higher than that in the control group and other groups, which indicates the effect of honey on increasing the total antioxidant capacity. Moreover, histological results of the present study indicate hepatic tissue damage, including hypertrophic degeneration and nuclear changes, enlargement of hepatic sinusoids, liver fibrosis and necrosis, and inflammation of hepatocytes in VPA groups compared to the control group and other groups. Findings of the study carried out with various doses of VPA showed hepatic lobular distortion in the rats treated with doses of 300 mg/kg/day and above. Furthermore, aggregations of nuclei and necrosis were also observed in 300 mg/kg VPA [21]. Several studies have indicated liver steatosis and necrosis induced by VPA [22]. However, the study conducted by Keterson et al. did not confirm the pathological effects at doses of 700 mg/kg/day [23]. In another study, an increase in liver weight (due to the excess production of collagen) and hepatic lesions had occurred in the VPA treatment group at a dose of 250 mg/kg [14]. In the research carried out by Noha et al., the histopathological examination of VPA-treated rats showed alterations in the normal liver tissue, hepatocyte degeneration, and inflammatory cell infiltration. This work suggests that oxidative stress due to VPA triggers inflammation cascade and transcription factors [24]. These findings were consistent with the results of our study. According to the studies mentioned above and our findings, it appears that the long-term use of VPA at different doses has shown some histological complications that finally lead to cell death, inflammation, and probably irreversible condition.

Estimation of liver enzymes was the other parameter that we measured to assess liver injury caused by VPA. The study conducted by Qiaoli et al. showed that the activities of the enzymes significantly increased in the dose-dependent manner [25]. Another study revealed that AST level did not show a significant increase above the dose of 300 mg/kg VPA. Since ALT is more specific to the liver than AST, elevation of this enzyme persists longer after hepatic damage, which may cause unequal outcomes in several studies. It is suggested that mild and reversible hepatic toxicity during VPA treatment is dose related and consists of changes in liver enzymes without clinical symptoms [24]. This article and similar cases probably need more in-depth investigations of various times and doses of VPA in rodent species to confirm their reports. We observed another phenomenon in the present study about liver enzymes. In the group treated only with VPA, the amount of AST was less than that in the first four groups. In TH plus VPA groups, increasing the TH dose resulted in a desirable outcome. Moreover, the levels of ALT enzyme presented significant differences and confusing outcomes among the groups. The level of this enzyme did not show significant enhancement in the VPA group compared to the control and TH groups. The findings related to GGT showed the highest level in the VPA group that was concurrent with some reports [24]. Our outcomes related to liver enzymes show lower amounts of ALT and AST and higher amount of GGT levels in the group treated with VPA. In this case, TH groups showed different levels of these enzymes which indicate the dose-dependent manner. Hence, it could be assumed that VPA and TH represent liver biochemical and hepatic tissue changes according to the infusion value.

Previous articles have shown that TH has a strong combination of antibacterial and antioxidant properties that can play significant roles in reducing lipid peroxidation. The inherent characteristics of honey include its antibacterial and antioxidant properties, which are due to the function of the enzyme glucose oxidase. This enzyme is inactive in full-density honey, but in diluted honey, it produces hydrogen peroxide and gluconic acid from activated glucose. Many natural antibacterial compounds have been identified from different types of honey, of which flavonoids are the main constituents. In the study conducted by Sanaa et al., the administration of high doses of royal jelly (as a natural antioxidant) alone caused mild inflammation [26]. Similarly, our study showed that concomitant use of honey in different doses after VPA administration reduces tissue inflammatory changes. Al-Yahya et al. found that consumption of honey, along with liver toxicity, could be effective in reducing inflammation and levels of liver enzymes [27]. Prescribing honey can help maintain the balance of the body's antioxidant system after an increase in ROS. As in the G6 group, concomitant use of TH (2 g) and VPA reduced the amount of liver enzymes and inflammation. However, in G6 and G8 groups, despite the administration of TH with medication, the effects of inflammation were observed. This indicates that different doses of honey can have different effects on reducing or increasing hepatic complications caused by VPA. In the study by Kassi et al., it has been confirmed that thyme honey has a unique monoterpene which shows apoptosis activity in cancer cells [28]. Findings of the study on natural honey suggest that honey may have protective effects by decreasing AST, ALT, and GGT levels after inducing toxicity via aflatoxicosis. This study also illustrated the supplementary role of honey in histological changes, decreasing the MDA level, inhibiting lipid peroxidation, and scavenging free radicals [29]. The royal jelly and honey significantly restored the changes of liver enzymes caused by cisplatin injection due to its antioxidant influence as a free radical scavenger [30]. Furthermore, some investigators have reported that honey has a protective role in reducing the disturbances in liver function caused by the consumption of chemical drugs. The findings of previous studies and the present research confirm the efficacy of honey administration in counteracting with toxic materials. However, it is important to note that special doses of natural antioxidants may be effective in reducing hepatic failure.

## 5. Conclusion

Fatal hepatotoxicity is one of the most important side effects of valproic acid. This complication occurs following the process of oxidative stress in the liver tissue. One of the strategies that can reduce these injuries is the use of antioxidants. Based on the results of this study, it seems that TH has been able to improve these liver complications and reduce the rate of liver cell destruction.

## Figures and Tables

**Table 1:** Standard formula for calculating MDA.

*y* = *Ax* + *B*	*y* = 0.000417142857142857*x* + 0.242920634920635
*A*	0.000417143
*B*	0.242920635
*x*	Concentration
*y*	OD
ml	0.02
*A*/mg or ml	0.020857143

Determination of sample purity with wavelength absorption level. OD: optical density.

**Table 2:** Standard formula for calculating TAC.

*y* = *Ax* + *B*	*y* = 0.178952380952381*x* + 0/188746031746032
*A*	0.178952381
*B*	0.188746032
*x*	Concentration
*y*	OD

**Table 3:** Histological scoring comparison of the groups.

Hepatocyte changes	G1	G2	G3	G4	G5	G6	G7	G8
Hypertrophic degeneration of hepatocytes' nuclei	-	-	-	-	++++	+++	++	+++
Expansion of hepatocyte sinusoids	-	-	+	+	++++	+++	++	+++
Hepatic fibrosis and necrosis	-	-	+	+	++++	+++	+	++
Inflammation	-	+	+	+	++++	+++	+	+++
Kupffer cell hypertrophy	-	+	+	+	+++	++	++	+++

-: no changes; +: minimum; ++: mild; +++: medium; ++++: severe.

## Data Availability

This data if necessary after revising would be placed.

## Abbreviations

ALP: Alkaline phosphatase
AST: Aspartate amino transferase
ALT: Alanine amino transferase
LDH: Lactate dehydrogenase
GGT: Gamma glutamyl transferase
VPA: Valproic acid
MDA: Malondialdehyde
TAC: Total antioxidant capacity
AlCl: Aluminum chloride
DPPH: 2,2-Diphenyl-1-picrylhydrazyl
SOD: Superoxide dismutase
ROS: Reactive oxygen species.
